# Acute versus chronic phase mechanisms in a rat model of CRPS

**DOI:** 10.1186/s12974-015-0472-8

**Published:** 2016-01-19

**Authors:** Tzuping Wei, Tian-Zhi Guo, Wen-Wu Li, Wade S. Kingery, John David Clark

**Affiliations:** Physical Medicine and Rehabilitation Service, (RM A-132), Veterans Affairs Palo Alto Health Care System, 3801 Miranda Avenue, Palo Alto, CA 94304 USA; Anesthesiology Service, Veterans Affairs Palo Alto Health Care System, 3801 Miranda Avenue (112-A), Palo Alto, CA 94304 USA; Department of Anesthesiology, Stanford University School of Medicine, Stanford, CA 94304 USA

**Keywords:** Fracture, Complex regional pain syndrome, NK1 receptor, Cytokines, Nerve growth factor, Immobilization

## Abstract

**Background:**

Tibia fracture followed by cast immobilization in rats evokes nociceptive, vascular, epidermal, and bone changes resembling complex regional pain syndrome (CRPS). In most cases, CRPS has three stages. Over time, this acute picture, allodynia, warmth, and edema observed at 4 weeks, gives way to a cold, dystrophic but still painful limb. In the acute phase (at 4 weeks post fracture), cutaneous immunological and NK1-receptor signaling mechanisms underlying CRPS have been discovered; however, the mechanisms responsible for the chronic phase are still unknown. The purpose of this study is to understand the mechanisms responsible for the chronic phases of CRPS (at 16 weeks post fracture) at both the peripheral and central levels.

**Methods:**

We used rat tibial fracture/cast immobilization model of CRPS to study molecular, vascular, and nociceptive changes at 4 and 16 weeks post fracture. Immunoassays and Western blotting were carried out to monitor changes in inflammatory response and NK1-receptor signaling in the skin and spinal cord. Skin temperature and thickness were measured to elucidate vascular changes, whereas von Frey testing and unweighting were carried out to study nociceptive changes. All data were analyzed by one-way analysis of variance (ANOVA) followed by Neuman-Keuls multiple comparison test to compare among all cohorts.

**Results:**

In the acute phase (at 4 weeks post fracture), hindpaw allodynia, unweighting, warmth, edema, and/or epidermal thickening were observed among 90 % fracture rats, though by 16 weeks (chronic phase), only the nociceptive changes persisted. The expression of the neuropeptide signaling molecule substance P (SP), NK1 receptor, inflammatory mediators TNFα, IL-1β, and IL-6 and nerve growth factor (NGF) were elevated at 4 weeks in sciatic nerve and/or skin, returning to normal levels by 16 weeks post fracture. The systemic administration of a peripherally restricted IL-1 receptor antagonist (anakinra) or of anti-NGF inhibited nociceptive behaviors at 4 weeks but not 16 weeks. However, spinal levels of NK1 receptor, TNFα, IL-1β, and NGF were elevated at 4 and 16 weeks, and intrathecal injection of an NK1-receptor antagonist (LY303870), anakinra, or anti-NGF each reduced nociceptive behaviors at both 4 and 16 weeks.

**Conclusions:**

These results demonstrate that tibia fracture and immobilization cause peripheral changes in neuropeptide signaling and inflammatory mediator production acutely, but central spinal changes may be more important for the persistent nociceptive changes in this CRPS model.

## Background

Complex regional pain syndrome type I (CRPS) is an often chronic pain condition characteristically disproportionate to the inciting event. The syndrome develops after a range of injuries including fractures, soft tissue trauma to the extremities, or as a consequence of a separate disease process like stroke or myocardial infarction [1]. In most cases, CRPS has three stages, but CRPS does not always follow this pattern. In many patients, the early symptoms are of a warm, erythematous, swollen, painful limb, the so-called “warm phase” thought to be supported by neurogenic inflammation [2–4]. In the acute phase, cutaneous immunological mechanisms underlying CRPS have been discovered, including autoimmunity [5], keratinocyte activation, proliferation, and expression of inflammatory mediators such as tumor necrosis factor alpha (TNFα), interleukin-1 beta (IL-1β) and interleukin-6 (IL-6), nerve growth factor (NGF), and mast cell activation [6, 7]. Substance P (SP), acting through up-regulated neurokinin 1 (NK1) receptors expressed in the peripheral tissues of the involved limb, appears to be a key signaling molecule supporting the signs and symptoms of CRPS [8, 9]. Over time, however, this acute picture gives way to a cold, dystrophic but still painful limb. Changes with origins clearly within the central nervous system (CNS) such as emotional problems, cognitive changes, and movement disorders can be observed in some patients [10, 11]. Prospective studies have observed a gradual spontaneous resolution of CRPS symptoms and signs in distal limb fracture cases, with 66 to 80 % of cases completely resolving by 6 months after injury [12–15]. The mechanisms supporting the chronic phases of CRPS are still very poorly understood.

The fracture/cast immobilization rodent model of CRPS displays the principal signs of CRPS including warmth, edema, enhanced neurogenic extravasation, epidermal hypertrophy, bone loss, and nociceptive changes [16–19]. These animals also show an evolution of signs over time to resemble the more chronic phases of CRPS in humans [17]. Using this model, it has been shown that neuropeptide signaling is particularly important for nociceptive sensitization and cytokine generation in the affected limb 4 weeks after fracture when acute phase changes are present. However, it is unclear whether these peripheral mechanisms continue to contribute to the persistent signs of CRPS in the chronic phases of the model, or whether central changes become the predominant mechanistic factors. Some evidence from CRPS patients suggests that peripheral inflammatory mechanisms may fade with time including levels of skin cytokines and mast cell abundance in skin [6, 20].

Therefore, in the present study, we hypothesized that the enhanced vascular permeability, edema, warmth, and nociceptive sensitization observed in the rodent CRPS model could be attributed to enhanced peripheral neuropeptide and cytokine signaling in the acute phase, whereas the persistent allodynia observable 16 weeks post fracture would be attributable to NK1 receptor activation and neuroinflammation in the spinal cord. Such findings might enhance our understanding of the evolution of CRPS over time and may suggest approaches to tailoring CRPS treatment to its duration and clinical features.

## Methods

These experiments were approved by our institute’s Subcommittee on Animal Studies and followed the guidelines of the International Association for the Study of Pain (IASP) [21]. Adult (9 months old) male Sprague Dawley rats (Simonsen Laboratories, Gilroy, CA, USA) were used in all experiments. The animals were housed individually in isolator cages with solid floors covered with 3 cm of soft bedding and were fed and watered ad libitum. During the experimental period, the animals were fed Teklad Global 18 % Protein Rodent Diet (www.Envigo.com, Madison, WI.) and were kept under standard conditions with a 12-h light–dark cycle. For all experiments, animals were randomly assigned to study groups, and observers were blinded to treatment conditions such as drug injections.

### Surgery and recovery

Tibial fracture was performed under isoflurane anesthesia as we have previously described [17]. The right hindlimb was wrapped in stockinet (2.5 cm wide), and the distal tibia was fractured using pliers with an adjustable stop that had been modified with a 3-point jaw. The hindlimb was wrapped in casting tape so the hip, knee, and ankle were flexed. The cast extended from the metatarsals of the hindpaw up to a spica formed around the abdomen. To prevent the animals from chewing at their casts, the cast material was wrapped in galvanized wire mesh. The rats were given subcutaneous saline and buprenorphine (0.03 mg/kg) immediately after the procedure and on the next day after fracture for postoperative hydration and analgesia. At 4 weeks, the rats were anesthetized with isoflurane, and the cast removed with a vibrating cast saw. All rats used in this study had mechanical union at the fracture site after 4 weeks of casting. To study changes in the chronic phase, after cast was removed at 4 weeks, fracture rats were allowed to recover in cages until 16 weeks after fracture prior to behavioral and biochemical testing.

### Drugs

The NK1 receptor antagonist LY303870 was a generous gift from Dr. L. Phebus (Eli Lily Company, Indianapolis, IN, USA). This compound has nanomolar affinity for the rat NK1 receptor, has no affinity for 65 other receptors and ion channels, has no sedative, cardiovascular or core body temperature effects in rats at systemic doses up to 30 mg/kg, and is physiologically active for 24 h after a single systemic dose of 10 mg/kg [22–24]. The doses of systemic and intrathecal LY303870 used in the study were based on our prior studies demonstrating analgesic efficacy with these doses in the 4-week post-fracture rat model [17, 25]. LY303870 (30 mg/kg/day, i.p.) was administered daily for 7 days and one final dose at 1 h prior to testing [24] or via intrathecal injection (10 μl at 20 μg/μl, 30 min prior to testing). All testing was carried out at 4 and 16 weeks after tibia fracture.

A naturally occurring IL-1-receptor antagonist (IL-1ra) modulates the activity of IL-1β in vivo. Anakinra, a recombinant met-human IL-1ra (r-metHuIL-1ra, Kineret, Amgen) that binds to the IL-1 type I receptor (IL-1 RI), effectively inhibits IL-1β signal transduction [26]. To test the hypothesis that IL-1β signaling mediates the vascular and nociceptive changes observed after tibia fracture in rats, osmotic pumps (model 2ML4, Alzet) were used for delivering anakinra IL-1ra. The pumps were inserted subcutaneously over the rat’s back. Based on previous studies using IL-1ra in rodents [27–29] and based on discussions with lead scientists from Amgen, it was previously decided to administer 10 mg/kg/day of anakinra via subcutaneous pump for 28 days prior to testing at 4 weeks post fracture. During the present study, we discovered that, at 16 weeks, systemic treatments with LY303870 and anti-NGF lost more than 50 % of their anti-nociceptive effects observed at 4 weeks post fracture. In addition, skin cytokines at 16 weeks, including IL-1β, declined from 4 weeks post fracture, but spinal cord cytokine concentrations at 16 weeks remained high and close to the peak concentration at 4 weeks post fracture. These data suggested that SP signaling and inflammation in the spinal cord at the central level might contribute to nociceptive abnormalities at 16 weeks. To demonstrate the effects of anakinra on fracture-induced nociceptive abnormalities at the spinal cord level, anakinra was administered via a single intrathecal injection (10 μl at 10 μg/μl) at 1 h prior to testing at 4 and 16 weeks post fracture. As a comparison at the systemic level, 100 mg anakinra/kg, one dose i.p. was injected 1 h prior to testing at 16 weeks post fracture. All testing was carried out at 4 and 16 weeks after tibia fracture.

The anti-NGF antibody muMab 911 (Rinat Laboratories, Pfizer Inc.) is a TrkA immunoglobulin G (TrkA-IGG) fusion molecule that binds to the NGF molecule, thus blocking the binding of NGF to the TrkA and p75 NGF receptors and inhibiting TrkA autophosphorylation [30]. Pharmacokinetic and behavioral experiments in rodents indicate that muMab 911 has a terminal half-life of 5 to 6 days in plasma and that a 10 mg/kg dose administered every 5 or 6 days reduces nociceptive behavior in a variety of rodent chronic pain models [31–34]. Based on these data and discussions with lead scientists at Rinat, it was determined to administer muMab 911 via a single subcutaneous injection (10 mg/kg) 7 days prior to testing or via a single intrathecal injection (10 μl at 1.24 μg/μl) 1 h prior to testing. All testing was carried out at 4 and 16 weeks after tibia fracture.

### Hindpaw nociception

To measure mechanical allodynia in the rats, an up-down von Frey testing paradigm was used as we have previously described [17, 35, 36]. Rats were placed in a clear plastic cylinder (20 cm in diameter) with a wire mesh bottom and allowed to acclimate for 15 min. The paw was tested with one of a series of eight von Frey hairs ranging in stiffness from 0.41 to 15.14 g. The von Frey hair was applied against the hindpaw plantar skin at approximately midsole, taking care to avoid the tori pads. The fiber was pushed until it slightly bowed. Hindpaw withdrawal from the fiber was considered a positive response. The initial fiber presentation was 2.1 g, and the fibers were presented according to the up-down method of Dixon to generate six responses in the immediate vicinity of the 50 % threshold. Stimuli were presented at an interval of several seconds. In addition, to measure hindpaw unweighting, an incapacitance device (IITC Inc. Life Science, Woodland, CA, USA) was used. The rats were manually held in a vertical position over the apparatus with the hindpaws resting on separate metal scale plates, and the entire weight of the rat was supported on the hindpaws. The duration of each measurement was 6 s, and ten consecutive measurements were taken at 60-s intervals. Eight readings (excluding the highest and lowest ones) were averaged to calculate the bilateral hindpaw weight-bearing values.

### Hindpaw temperature

The room temperature was maintained at 23 °C, and humidity ranged between 25 and 45 %. The temperature of the hindpaw was measured using a fine wire thermocouple (Omega, Stanford, CT, USA) applied to the paw skin, as previously described [17, 35, 36]. The investigator held the thermistor wire using an insulating Styrofoam block. Three sites were tested over the dorsum of the hindpaw; the space between the first and second metatarsals (medial), the second and third metatarsals (central), and the fourth and fifth metatarsals (lateral). After a site was tested in one hindpaw, the same site was immediately tested in the contralateral hindpaw. The testing protocol was medial dorsum right then left, central dorsum right then left, lateral dorsum right then left, medial dorsum left then right, central dorsum left then right, and lateral dorsum left then right. The six measurements for each hindpaw were averaged for the mean temperature.

### Hindpaw thickness

A laser sensor technique was used to determine the dorsal-ventral thickness of the hindpaw, as we have previously described [35]. For laser measurements, each rat was briefly anesthetized with isoflurane and then held vertically so the hindpaw rested on a table top below the laser. The paw was gently held flat on the table with a small metal rod applied to the top of the ankle joint. Using optical triangulation, a laser with a distance measuring sensor was used to determine the distance to the table top and to the top of the hindpaw at a spot on the dorsal skin over the midpoint of the third metatarsal, and the difference was used to calculate the dorsal-ventral paw thickness. The measurement sensor device used in these experiments (4381 Precicura, Limab, Goteborg, Sweden) has a measurement range of 200 mm with a 0.01-mm resolution.

### Epidermal thickness

This experiment determined whether fracture chronically increases epidermal thickness. four and sixteen-week post-fracture rats were anesthetized with isoflurane and then transcardially perfused with 4 % paraformaldehyde in phosphate-buffered saline (PBS, pH 7.4); the dorsal hindpaw skin including sub-dermal layers was immediately removed and post-fixed in 4 % paraformaldehyde (PFA) for 2 h; then, the tissues were treated with 30 % sucrose in PBS at 4 °C before embedding in OCT. Following embedding, 20-μm slices were made using a cryostat and mounted onto Superfrost microscope slides (Fisher scientific, Pittsburgh, PA). All sections were stored at −70 °C until use for immunohistochemistry. Frozen sections were permeabilized and blocked with PBS containing 10 % donkey serum and 0.3 % Triton X-100 prior to primary antibody incubation. Sections were incubated with a pan-keratinocyte marker recognizing both the acidic and basic (types I and II) subfamilies of cytokeratins for keratinocyte labeling, and fluorescent immunohistochemistry and confocal microscopy were performed as previously described [19]. Epidermal thickness measurements were made using LSM Image Browser from Zeiss LSM Data Server. A blinded observer selected four to eight sections from each skin specimen, and four to eight thickness measurements were made in each section to derive a mean score for that sample. The individual mean scores were then used to calculate the mean thickness and standard error of the mean for the control limbs and the fracture limbs at 4 and 16 weeks post injury.

### Homogenization procedure and enzyme immunoassay for TNFα, IL-1β, IL-6, and NGF

Rat hindpaw dorsal skin and ipsilateral spinal cord of lumbar enlargement section were collected after behavioral testing or at time points as indicated and frozen immediately on dry ice. Skin and spinal cord tissues were cut into fine pieces in ice-cold phosphate-buffered saline (PBS), pH 7.4, containing protease inhibitors (aprotinin [2 μg/ml], leupeptin [5 μg/ml], pepstatin [0.7 μg/ml], and PMSF [100 μg/ml]; Sigma, St. Louis, MO, USA) followed by homogenization using a rotor/stator homogenizer. Homogenates were centrifuged for 5 min at 14,000 *g*, and at 4 °C. Supernatants were transferred to fresh pre-cooled Eppendorf tubes. Triton X-100 (Boehringer Mannheim, Germany) was added at a final concentration 0.01 %. The samples were centrifuged again for 5 min at 14,000 *g* at 4 °C. The supernatants were aliquoted and stored at −80 °C. TNFα, IL-1β, and IL-6 protein levels were determined using EIA kits (R&D Systems, Minneapolis, MN, USA). The NGF concentrations were determined using the NGF Emax® ImmunoAssay System kit (Promega, Madison, WI, USA) according to the manufacturer’s instructions. The optical density (OD) of the reaction product was read on a microplate reader at 450 nm. The concentrations of TNFα, IL-1β, IL-6, and NGF proteins were calculated from the standard curve at each assay. Positive and negative controls were included in each assay. Each protein concentration was expressed as picogram per milligram total protein. Total protein contents in all tissue extracts were measured by the Coomassie Blue Protein Assay Kit (Bio-Rad, Hercules, CA).

### Enzyme immunoassay procedure for sciatic nerve SP

The aim of this experiment was to determine whether fracture induced up-regulated SP protein expression in the sciatic nerve at 4 and 16 weeks post fracture. The right sciatic nerve was collected under isoflurane anesthesia, immediately frozen, and weighed. Nerve samples were minced in 1 ml of 3:1 ethanol/0.7 M HCl and homogenized for 20 s. The homogenates were shaken for 2 h at 4 °C and centrifuged at 3000 *g* for 20 min at 4 °C. The supernatant was frozen and lyophilized, and the lyophilized product was stored at −80 °C. All nerve samples were assayed in duplicate using an EIA kit to determine SP levels (Assay Designs, Ann Arbor, MI) following the manufacturer’s protocols.

### SP-facilitated extravasation in fracture rats

This experiment tested the hypothesis that tibia fracture facilitates SP-evoked extravasation responses in the injured hindlimb at 4 and 16 weeks after injury, when compared with the normal controls. Five minutes after injection of Evans blue dye (50 mg/kg in Ringers, Sigma), SP (10 μg/kg, Sigma) was injected intravenously into the internal jugular vein. Five minutes after SP injection, the rats were anesthetized with isoflurane, transcardially perfused as previously described, and the plantar and dorsal skin on each hindpaw was collected for dye content determination [16].

### Western blotting

These experiments tested the hypothesis that tibia fracture with cast immobilization can induce chronic increases in the NK1-receptor protein in the hindpaw skin and spinal cord of lumbar enlargement. At 4 or 16 weeks after fracture, the ipsilateral hindpaw dorsum skin was collected under isoflurane anesthesia and was homogenized in modified RIPA buffer (50 mM Tris–HCl, 150 mM NaCl, 1 mM EDTA, 1 % Igepal CA-630, 0.1 % SDS, 50 mM NaF, and 1 mM NaVO3) containing protease inhibitors (aprotinin [2 μg/ml], leupeptin [5 μg/ml], pepstatin [0.7 μg/ml], and PMSF [2 mM]; Sigma, St. Louis, MO, USA). The homogenate was centrifuged at 13,000*g* for 30 min at 4 °C. Total protein concentration of the homogenate was measured using a Coomassie Blue Protein Assay (Bio-Rad, Hercules, CA) and normalized against BSA protein standards (Pierce, Rockford, IL). The supernatant was subjected to Western blot analysis using our previously described methods [16] to elucidate changes in NK1-receptor protein expression in the skin or spinal cord. Equal amounts of protein (30 μg) were subjected to SDS-PAGE (12 % Tris–HCl acrylamide gel, Bio-Rad, Hercules, CA) and electrotransferred onto a polyvinylidene difluorided membrane (Millipore). The blots were blocked with 5 % non-fat dry milk in Tris-buffered saline, incubated with goat anti-rat NK1R primary antibody overnight at 4 °C and further incubated with HRP-conjugated secondary antibody (Santa Cruz Biotechnology, Santa Cruz, CA) for 1 h at room temperature. After washing in TBST three times, the blot was then incubated in ECL plus chemoluminescence reagents (Amersham, Piscataway, NJ) and scanned by PhosphoImager (Typhoon, GE Healthcare), and the band intensity was analyzed using ImageQuant 5.2 software (Molecular Dynamics, Piscataway, NJ) and normalized with the corresponding internal loading control band, β-actin. The specific protein/actin band intensity ratio represents the change of the specific protein.

### Study design

To study the acute and chronic changes in nociceptive behavior, vascular signs, and inflammatory responses, rats were randomly assigned to four primary experimental cohorts: 4-week controls, 4-week post fracture, 16-week controls, and 16-week post fracture. Control rats received no fracture or other treatments. At the designated time point, all cohorts were tested for bilateral hindpaw mechanical nociceptive withdrawal thresholds to von Frey fibers, weighting, hindpaw temperature, and hindpaw thickness as well as intravenous SP-induced hindpaw extravasation. Ipsilateral to the fracture, the hindpaw skin, as well as sciatic nerve and lumbar spinal cord was collected and stored for determination of TNFα, IL-1β, IL-6, and NGF protein, as well as SP and NK1 receptor levels, respectively.

Separate cohorts of fracture rats at the 4- and 16-week time points were treated systemically with vehicle (i.p. or s.c.), LY303870 (i.p.), anakinra (s.c. pump or i.p. injection), or anti-NGF (s.c. injection). Anakinra and anti-NGF are large molecules with poor CNS penetration; thus, we assumed that these treatments inhibited peripheral inflammatory mechanisms in the injured limb without affecting CNS inflammatory changes. Additional fracture rats at the 4- and 16-week time points were treated intrathecally with either vehicle, LY303870, anakinra, or anti-NGF to test the hypothesis that spinal cord SP signaling, IL-1β, and NGF contribute to pathological signs of CRPS in the acute and chronic phases. Each cohort was tested for bilateral hindpaw mechanical nociceptive withdrawal thresholds to von Frey fibers, unweighting, warmth, and edema.

### Statistical analysis

Statistical analysis was accomplished using a one-way analysis of variance (ANOVA) followed by Neuman-Keuls multiple comparison test to compare among all cohorts. All data are presented as the mean ± standard error (SE) of the mean, and differences are considered significant at a *P* value less than 0.05 (Prism 5, GraphPad Software, San Diego, CA, USA).

Hindpaw temperature, thickness, and mechanical nociceptive threshold data were analyzed as the difference between the fracture side (right, R) and the contralateral untreated side (left, L). Right hindpaw weight-bearing data were analyzed as a ratio between twice the right hindpaw weighting and the sum of the right (R) and left (L) hindpaw values ([2R/(R + L)] × 100 %).

## Results

### The time course of nociceptive, vascular, and trophic changes in the CRPS model

**Fig. 1:**
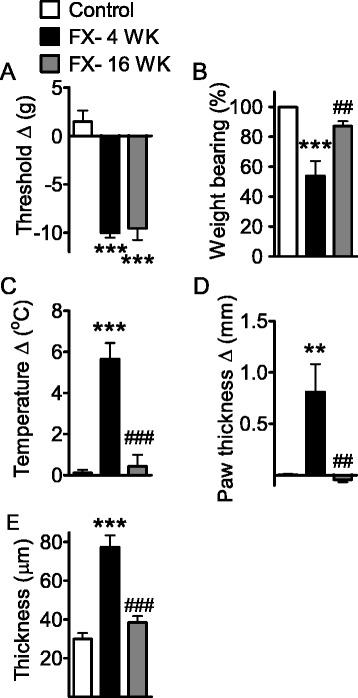
Differential recovery of nociceptive and vascular abnormalities in the rat CRPS. The measurements displayed were made at 4 and 16 weeks after fracture. **a** Tibia fracture-induced mechanical allodynia to von Frey testing. **b** Hindpaw unweighting. **c** Hindpaw warmth. **d** Hindpaw edema. **e** Epidermal thickness. Measurements for **a**–**d** represent the difference between the fracture ipsilateral side and the contralateral paw. The values displayed in panel **b** represent weight-bearing on the fracture hindlimb as a ratio to 50 % of bilateral hindlimb loading. **e** Changes in dorsal hindpaw epidermal thickness at 4 and 16 weeks after fracture. Immunostaining of keratin (a keratinocyte marker) was performed in dorsal hindpaw skin sections from control rats (*n* = 7), and from the ipsilateral hindpaws of fracture rats at 4 (*n* = 8) and 16 weeks (*n* = 8) post injury. Data are analyzed to compare fracture at 4 weeks versus no-fracture control cohorts and fracture at 16 weeks versus fracture at 4 weeks cohorts using one-way analysis of variance (ANOVA) followed by Neuman-Keuls multiple comparison test. *P < 0.05, **P < 0.01, and ***P < 0.001, fracture at 4 weeks (panels **a**–**d**, *n* = 6) versus control rats (panels **a**–**d**, *n* = 6); ^#^ *P* < 0.05, ^##^ *P* < 0.01, and ^###^ *P* < 0.001, fracture at 16 weeks (panels **a**–**d**, *n* = 5) versus fracture at 4 weeks cohorts

The nociceptive and vascular effects of tibia fracture followed by cast immobilization were examined in control and fracture rats at 4 weeks and at 16 weeks. Fracture-induced hindpaw mechanical allodynia at 4 weeks post fracture, and subsequently, more than 90 % of the allodynia persisted (Fig. 1a) at 16 weeks. In contrast, most fracture-induced hindlimb unweighting had recovered by 16 weeks, indicating different mechanisms may be responsible for these two nociceptive responses (Fig. 1b). Although only approximately 10 % unweighting deficit (2R*100 %/[R + L]) remained at 16 weeks, the actual bodyweight bearing deficit (R-L, with average body weight approximately 500 g) was very significant as in pain. Fracture-induced hindpaw vascular abnormalities at 4 weeks, including warmth (5.65 °C increase in skin temperature, Fig. 1c), and edema (hindpaw thickness increased by 0.8 mm, Fig. 1d). These hindpaw vascular changes subsided completely by 16 weeks. Similarly, epidermal thickness was increased at 4 weeks post fracture (47 μm increase in hindpaw epidermal thickness), but not at 16 weeks (Fig. 1e).

### Increased cutaneous cytokine and neurotrophin levels at 4 weeks but not 16 weeks post fracture

**Fig. 2:**
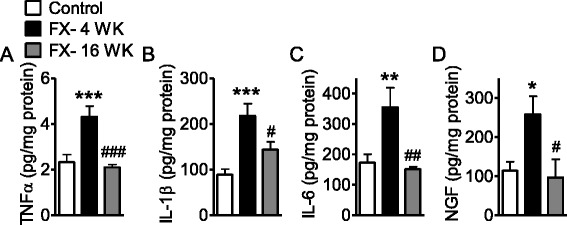
Cytokine and NGF levels in hindpaw skin were elevated at 4 weeks and returned to normal levels by 16 weeks after fracture. TNFα (**a**), IL-1β (**b**), IL-6 (**c**) and nerve growth factor (NGF) (**d**) levels in hindpaw skin was measured by specific EIAs at 4 and 16 weeks after fracture. Data are expressed as mean values (pg/mg protein) ± standard error (SE) and analyzed using one-way ANOVA followed by Neuman-Keuls multiple comparison test to compare different time points. *P < 0.05, **P < 0.01, and ***P < 0.001 versus control; ^#^ *P* < 0.05, ^##^ *P* < 0.01, and ^###^ *P* < 0.001 versus fracture at 4 weeks, (*n* = 8 per cohort)

Increased levels of the cytokines TNFα, IL-1β, IL-6, and the neurotrophin NGF were observed in the hindpaw skin of the affected limb at 4 weeks after fracture (Fig. 2a–d). All of these cutaneous inflammatory changes had returned to baseline by the 16-week time point.

### Increased cutaneous SP signaling at 4 weeks but not 16 weeks after fracture

**Fig. 3:**
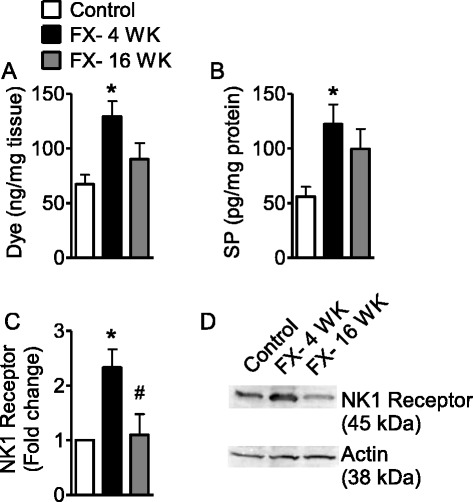
Fracture-facilitated SP-induced Evan’s blue extravasation at 4 weeks but not 16 weeks. **a** Evan’s blue dye extravasation in rats (control cohort, *n* = 5; FX- 4 WK, *n* = 5; FX- 16 WK, *n* = 7) after SP injection. **b** Fracture induced SP expression in sciatic nerve tissue at 4 and 16 weeks after fracture (*n* = 8 per cohort). **c** Fracture-induced NK1-receptor expression in hindpaw skin at 4 and 16 weeks after fracture (*n* = 3 per cohort). **d** Representative Western blot of skin NK1 receptor and actin. Data are expressed as mean values (pg/mg protein or fold change) ± standard error (SE) and analyzed using one-way ANOVA followed by Neuman-Keuls multiple comparison test to compare control and fracture cohorts at different time points. *P < 0.05, **P < 0.01, and ***P < 0.001 versus control; ^#^ *P* < 0.05, ^##^ *P* < 0.01, and ^###^ *P* < 0.001 versus fracture at 4 weeks

Previously, we reported enhanced SP-evoked extravasation as a result of post-junctional facilitation of SP signaling in the fracture model at the 4-week time point [16]. Here, we observed, similar to previous data, that SP intravenous injection caused significantly more Evan’s blue extravasation at 4 weeks post fracture than in control rats (Fig. 3a). However, this effect subsided by 16 weeks. In addition, fracture induced a 119 % increase in SP expression in the sciatic nerve at 4 weeks, but SP levels had declined by 20 % at 16 weeks post fracture (Fig. 3b). In addition, NK1-receptor expression in the skin increased at 4 weeks post fracture but returned to control levels at 16 weeks (Fig. 3c, d).

### Systemic treatment with LY303870, anakinra, anti-NGF reversed fracture-induced nociceptive and vascular changes at 4 weeks after fracture

**Fig. 4:**
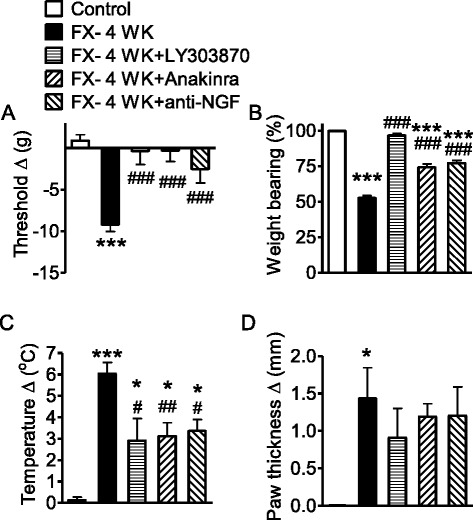
Systemic administration of LY303870, anakinra, and anti-NGF reversed fracture-induced nociceptive and vascular responses at 4 weeks after fracture. LY303870 (30 mg/kg/day i.p.) was administered for 7 days between 3 and 4 weeks post fracture. Anakinra was administered at 10 mg/kg/day s.c. in a pump for 28 days prior to testing at 4 weeks post fracture. Anti-NGF (10 mg/kg s.c.) was administered at 7 days prior to testing at 4 weeks post fracture. **a** Tibia fracture induced mechanical allodynia to von Frey testing at 4 weeks (control cohort, *n* = 10; FX- 4 WK, *n* = 10), and LY303870 (*n* = 8), anakinra (*n* = 10), and anti-NGF (n = 10) all reduced this sensitization. **b** At 4 weeks, unweighting developed in the ipsilateral hindpaw in fracture rats, and again, the test agents reduced the unweighting response. **c** Warmth was observed in fracture rats at 4 weeks, but fracture rats treated with LY303870, anakinra, or anti-NGF showed reduced temperature differences. **d** Edema indicated as an increase in hindpaw thickness was found at 4 weeks after fracture. Cohorts of rats treated with each of the test agents failed to show statistically significant changes in edema. Measurements for **a**, **c**, and **d** represent the difference between the fracture ipsilateral side and the contralateral paw. Measurements displayed in **b** represent weight-bearing on the fracture hindlimb as a ratio to 50 % of bilateral hindlimb loading. Data are expressed as mean values ± standard error (SE) and analyzed to compare fracture at 4 weeks versus non-fracture control cohorts and fracture with treatments versus fracture at 4 weeks cohorts using one-way ANOVA followed by Neuman-Keuls multiple comparison test. *P < 0.05, **P < 0.01, and ***P < 0.001, fracture at 4 weeks versus control rats; ^#^ *P* < 0.05, ^##^ *P* < 0.01, and ^###^ *P* < 0.001, fracture with treatments versus fracture at 4 weeks (panels **b**–**d**, control cohort, *n* = 6; FX- 4 WK, *n* = 8; FX- 4 WK + LY303870, *n* = 8; FX- 4 WK + anakinra, *n* = 10; FX- 4 WK + anti-NGF, *n* = 7)

**Fig. 5:**
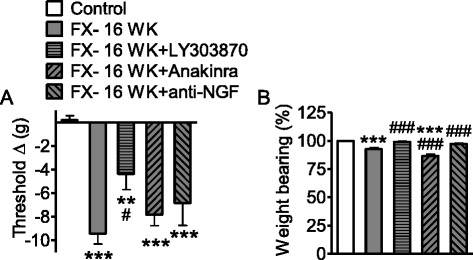
Systemic administration of LY303870, anakinra, and anti-NGF poorly reversed fracture-induced nociceptive responses at 16 weeks. Vehicle, LY303870, anakinra, or anti-NGF was administered between 15 and 16 weeks post fracture, and the rats were tested at 16 weeks post fracture. LY303870 (same treatment as Fig. 4) was injected for 7 days prior to testing, anakinra (100 mg/kg i.p.) was injected once at 1 h prior to testing, and anti-NGF (same treatment as Fig. 4) was injected once at 7 days prior to testing at 16 weeks post fracture. **a** Mechanical allodynia tested by von Frey fibers persisted to 16 weeks post fracture. LY303870, but not anakinra or anti-NGF, reversed fracture-induced allodynia at the 16-week time point. **b** Approximately 10 % unweighting remained at 16 weeks post fracture. Both LY303870 and anti-NGF reversed this remaining hindpaw unweighting. Data are expressed as mean values ± standard error (SE) and analyzed to compare fracture at 16 weeks versus non-fracture control cohorts and fracture with treatments versus fracture at 16 weeks cohorts using one-way ANOVA followed by Neuman-Keuls multiple comparison test. *P < 0.05, **P < 0.01, and ***P < 0.001 versus control rats; ^#^ *P* < 0.05, ^##^ *P* < 0.01, and ^###^ *P* < 0.001, fracture with treatments versus fracture at 16 weeks (control cohort, *n* = 10; FX- 4 WK, *n* = 8–10; FX- 4 WK + LY303870, *n* = 8–9; FX- 4 WK + anakinra, *n* = 6; FX- 4 WK + anti-NGF, *n* = 6–8)

To assess SP, IL-1β, and NGF-mediated contributions to the nociceptive and vascular changes in this model at 4 (Fig. 4) and 16 weeks (Fig. 5) after fracture, rats were systemically treated with the NK1-receptor antagonist LY303870 (30 mg/kg/day i.p. for 7 days and one additional last dose given at 1 h prior to testing), the IL-1β inhibitor anakinra (via a pump at 10 mg/kg/day for 28 days, s.c.), or the NGF inhibitor anti-NGF (one dose s.c. injection, 10 mg/kg at 7 days prior to testing). The administration of LY303870 or anakinra to 4-week post-fracture rats effectively abolished hindpaw allodynia (Fig. 4a). In addition, anti-NGF also reduced allodynia by 70 %. A similar trend was found in reversing unweighting by LY303870, anakinra, and anti-NGF antibody (Fig. 4b). The NK1-receptor antagonist, LY303870, completely reversed unweighting, and the IL-1β and NGF inhibitors each significantly reduced the unweighting response in the rats at 4 weeks after fracture. All three agents reduced the temperature difference between the hindpaws of the 4-week fracture rats (Fig. 4c), with the effect size ranging from 30 to 50 %. Tibia fracture also induced edema, indicated as an increase in hindpaw thickness of 1.44 mm in the ipsi- versus contralateral paws. The effects of LY303870, anakinra, and anti-NGF on edema did not reach statistical significancel, though there did appear to be strong trends towards reduced paw thickness.

### Systemic treatment with LY303870, anakinra, or anti-NGF had minimal effect on nociceptive and vascular changes at 16 weeks after fracture

**Fig. 6:**
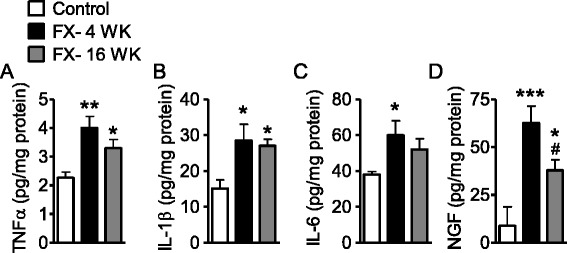
Cytokine and NGF levels in spinal cord tissue 4 and 16 weeks after fracture. The data displayed represent TNFα (**a**), IL-1β (**b**), IL-6 (**c**), and nerve growth factor (NGF) (**d**) levels in lumbar spinal cord tissue ipsilateral to fracture at 4 and 16 weeks post fracture. Data are expressed as mean values (pg/mg protein) ± standard error (SE) and analyzed using one-way ANOVA followed by Neuman-Keuls multiple comparison test to compare control and fracture cohorts at different time points. *P < 0.05, **P < 0.01, and ***P < 0.001 versus control; ^#^ *P* < 0.05, ^##^ *P* < 0.01, and ^###^ *P* < 0.001 versus fracture at 4 weeks (panels **a**, **c**, and **d**, *n* = 8 per cohort; panel **b**, *n* = 6 per cohort)

To study the effects of SP, IL-1β, and NGF signaling on nociceptive and vascular changes in the phase of the model resembling chronic CRPS, rats were fractured and casted for 4 weeks, followed by cast-removal and recovery for additional 12 weeks. When LY303870 (same dose as Fig. 4) and anti-NGF (same dose as Fig. 4) were administered between 15 and 16 weeks after fracture, only LY303870 showed a significant effect on von Frey allodynia (Fig. 5a), and the effect size was reduced dramatically compared with the 4-week results. At 16 weeks, systemic treatments with LY303870 and anti-NGF (Fig. 5a) lost more than 50 % of their anti-nociceptive effects observed at 4 weeks (Fig. 4a) post fracture. In addition, skin cytokine concentrations at 16 weeks, including IL-1β, declined from 4 weeks post fracture (Fig. 2), but all spinal cord cytokine concentrations at 16 weeks remained high (Fig. 6) and close to the peak concentration at 4 weeks post fracture. These data suggested that SP signaling and inflammation in the spinal cord at the central level might contribute to nociceptive abnormalities at 16 weeks. Thus, it became important to unveil the effects of these agents at the spinal level at 16 weeks. As shown in Fig. 5b, at 16 weeks, post-fracture hindpaw unweighting was approximately 10 %, and both LY303870 and anti-NGF had significant analgesic effects in unweighting. Vascular abnormalities had resolved by 16 weeks post fracture (Fig. 1). We did not undertake 28-day infusions of anakinra in the 16-week-fracture rats as we had at 4 weeks (Fig. 4), provided the younger rats were in cast for 4 weeks. Those animals would have been treated in a less chronic phase (12–16 weeks post fracture) by the time of assessment making data interpretation tenuous. We did, however, administer anakinra as a single i.p. bolus of 100 mg/kg at 1 h prior to testing at 16 weeks post fracture (Fig. 5) as a comparison to the treatment with anakinra via i.t. injection (Fig. 8c, d). At 16 weeks post fracture, anakinra i.t. (Fig. 8) showed an anti-nociceptive effect on allodynia and unweighting; however, anakinra i.p. did not (Fig. 5). Such a dose (anakinra, i.p. 100 mg/kg) was determined previously in a 4-week (acute stage)-fracture study in which 10 mg/kg/day of anakinra treatment was administered for 7 days prior to testing in a separate group of fracture rats. The 7-day treatment with anakinra yielded 50 % anti-allodynia and 50 % anti-unweighting effects at 4 weeks (data not shown) as compared to the 28-day treatment yielding 100 % anti-allodynia and 50 % anti-unweighting effects at 4 weeks (Fig. 4a, b). That is, a total dose of 70 mg over a 7-day period is sufficient to partially reduce fracture-induced nociceptive abnormalities at 4 weeks.

### Levels of cytokines, NGF, and NK1 receptor remain elevated in spinal cord tissue at 16 weeks after fracture

**Fig. 7:**
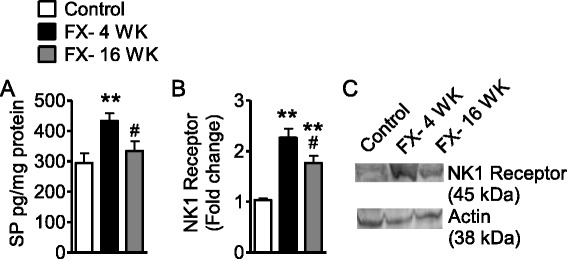
The elevation of substance P and NK1R levels in ipsilateral lumbar spinal cord tissue in fracture rats. **a** Substance P in spinal cord in control rats (*n* = 15) was elevated in response to fracture at 4 weeks (*n* = 14) but subsided by 16 weeks (*n* = 16) post fracture. **b** NK1-receptor (NK1R) levels were elevated in the lumbar enlargement ipsilateral spinal cord at 4 weeks by 2.2-fold and remained significantly elevated by 1.8-fold at 16 weeks post fracture (*n* = 3 per cohort). **c** The NK1R band intensities were normalized against actin band intensities. Data are expressed as mean values ± standard error (SE) and analyzed using one-way ANOVA followed by Neuman-Keuls multiple comparison test to compare control and fracture cohorts at different time points. *P < 0.05, **P < 0.01, and ***P < 0.001 versus control; ^#^ *P* < 0.05, ^##^ *P* < 0.01, and ^###^ *P* < 0.001 versus fracture at 4 weeks

At 16 weeks post fracture, nociceptive changes persisted while the effects of systemic treatments with LY303870, anakinra, and anti-NGF tended to lose analgesic efficacy. Anakinra and anti-NGF are large molecules with poor CNS penetration. Therefore, we hypothesized that nociceptive sensitization might be supported by persistent changes in these mediators at the spinal level. We therefore measured levels of the cytokines TNFα, IL-1β, and IL-6 as well as NGF in the spinal cord tissue of lumbar enlargement section ipsilateral to tibia fracture at 4 and 16 weeks post fracture. We found that the protein levels of all these mediators in spinal cord were significantly elevated at both 4 and 16 weeks post fracture, with the exception of IL-6 whose elevation at 16 weeks was not significant (Fig. 6). Only for NGF was there significantly less mediator present in spinal cord tissue at 16 versus 4 weeks (Fig. 6d). We went on to examine changes in SP/NK1 signaling molecules. At 4 weeks, both SP (Fig. 7a) and NK1-receptor (Fig. 7b) levels were elevated in spinal cord tissue ipsilateral to tibial fracture. While the elevation in SP content significantly normalized by 16 weeks, the elevation in expression of the NK1 receptor remained significant.

### Spinal NK1 receptor, IL-1β, and NGF signaling contribute to nociceptive changes in both the acute and chronic phases of the fracture model

**Fig. 8:**
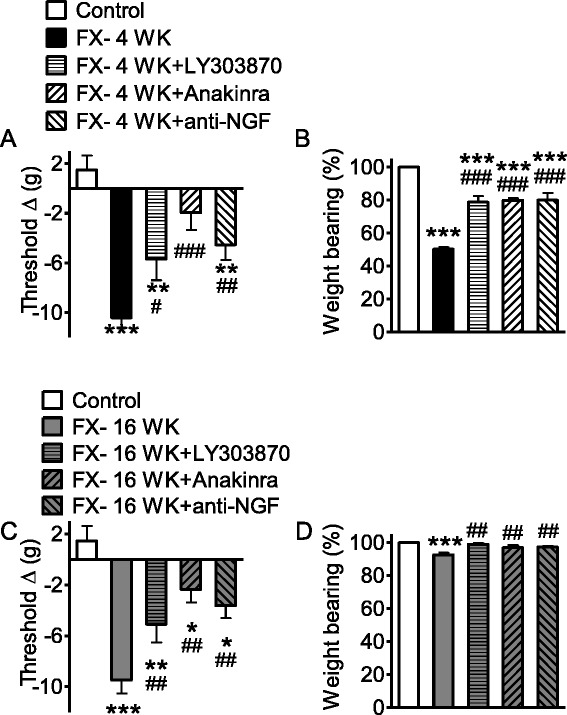
Intrathecal injections of LY303870, anakinra, and anti-NGF at 4 and 16 weeks post fracture reversed allodynia and unweighting. LY303870 (10 μl at 20 μg/μl, 30 min prior to testing), anakinra (10 μl at 10 μg/μl, 1 h prior to testing), and anti-NGF (10 μl at 1.24 μg/μl, 1 h prior to testing) were administered via intrathecal injection. **a** At 4 weeks, fracture induced allodynia and unweighting. Treatments with LY303870, anakinra, and anti-NGF reduced fracture-induced allodynia by 52, 84, and 62 %, respectively. **b** The same treatments reduced unweighting by 58, 60, and 60 %, respectively. **c** At 16 weeks, allodynia and unweighting were still significant. Treatments with LY303870, anakinra, and anti-NGF reduced fracture-induced allodynia by 54, 79, and 67 %, respectively. **d** The same treatments at 16 weeks post fracture reduced unweighting by 88, 61, and 67 %, respectively. Measurements for allodynia and unweighting were carried out and calculated as described for Fig. 1. Data are expressed as mean values ± standard error (SE) and analyzed using one-way ANOVA followed by Neuman-Keuls multiple comparison test to compare control and fracture at 4 weeks cohorts (control, *n* = 6; FX- 4 WK, *n* = 7; FX- 4 WK + LY303870, *n* = 7; FX- 4 WK + anakinra, *n* = 8; FX- 4 WK + anti-NGF, *n* = 8) as well as fracture with the treatment versus fracture cohorts at 16 weeks (control, *n* = 6; FX- 16 WK, *n* = 8; FX- 16 WK + LY303870, *n* = 8; FX- 16 WK + anakinra, *n* = 6; FX- 16 WK + anti-NGF, *n* = 8). *P < 0.05, **P < 0.01, and ***P < 0.001 versus control; ^#^ *P* < 0.05, ^##^ *P* < 0.01, and ^###^ *P* < 0.001 versus fracture at 4 or 16 weeks

Based on the evidence that NK1 receptor, cytokine, and neurotrophin levels remain elevated in spinal tissue throughout the 16-week time course of the fracture model, we next hypothesized that blocking the spinal effects of these signaling molecules might be effective in reducing nociceptive sensitization in both phases of the model. We therefore administered LY303870, anakinra, and anti-NGF intrathecally to the rats both 4 and 16 weeks after tibia fracture. The data presented in Fig. 8 show that each of these agents was effective in reducing hindpaw allodynia and hindpaw unweighting in both the acute (4 weeks) and chronic (16 weeks) phase rats at the spinal cord level, as opposed to the minimal systemic effects of anakinra and anti-NGF in the chronic phase (Fig. 5).

### The frequency of specific CRPS-like changes in the tibia fracture/cast immobilization model changes over time

**Fig. 9:**
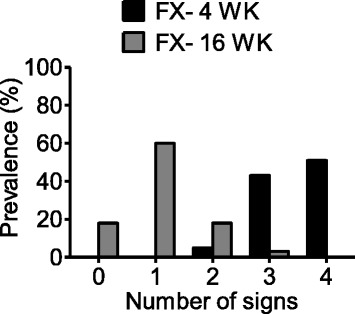
Prevalence of CRPS type I-like signs in the rat fracture model. At 4 weeks post fracture, (*n* = 37), 5, 43, and 51 % of fracture rats developed 2, 3, and 4 of the 4 signs, respectively, including allodynia, unweighting, warmth, and edema. At 16 weeks (*n* = 38), only 18 % of the fracture rats completely recovered, and 60 % of fracture rats still exerted at least 1 of the 4 CRPS type I-like signs

In these studies, we followed four CRPS-like changes manifest in the fracture rats similar to those used in the Budapest criteria for the diagnosis of CRPS [37]. Signs as well as criteria and prevalence of each sign at 4 and 16 weeks post fracture are listed in Table 1. Those changes included nociceptive (hindpaw allodynia, hindpaw unweighting) and vascular (warmth and edema) features. The data presented in Fig. 9 demonstrate that at 4 weeks post fracture, the majority of the rats developed three (43 %) to four (51 %) CRPS-like signs. At 16 weeks, only 18 % of the fracture rats completely recovered, and 60 % of fracture rats still exhibited at least one of the four CRPS-like signs. As shown in Table 1, allodynia was by far the most durable of the CRPS-like changes over time.

**Table 1:** Prevalence of CRPS-like signs (allodynia, unweighting, warmth, and edema) observed at 4 weeks and 16 weeks post fracture in fracture rats. The arbitrary criteria indicate approximately 30-50 % normalization of each measurement.

Signs	Allodynia	Unweighting	Warmth	Edema
Criteria	R-L difference	2R/(R + L) × 100 %	R-L difference	R-L difference
	Diagnostic threshold: 5-g reduction	Diagnostic threshold: 30 % reduction	Diagnostic threshold: 1 °C increase	Diagnostic threshold: 0.5-mm increase
FX- 4 WK	90 % (42/47)	87 % (33/38)	94 % (44/47)	73 % (32/44)
FX- 16 WK	72 % (34/47)	2 % (1/47)	19 % (9/47)	0 % (0/47)

*R* right hindpaw, *L* left hindpaw, *FX- 4 WK* 4 weeks after fracture, *FX- 16 WK* 16 weeks after fracture

## Discussion

Complex regional pain syndrome (CRPS) is a pain syndrome of modest prevalence in the overall population but has a relatively high incidence after injuries and surgeries to the limbs. Fracture of an extremity followed by immobilization is a particularly common set of circumstances leading to CRPS. The acute phase of the syndrome frequently involves an erythematous, warm, swollen, painful limb, all signs of acute neurogenic inflammation [38]. While some patients retain these signs of inflammation, it is more common for the warmth and swelling to resolve over the course of months with a normal or even cool atrophic but still painful limb in the chronic phases of the condition [39]. Thus CRPS is often thought to have an acute or “warm” phase followed by a more chronic “cold” phase. This difference is seldom considered when providing therapeutic interventions, though the mechanisms supporting the condition appear to be changing. Our earlier studies showed that gene transcription patterns within the CNS change as fracture mice progress from the acute to the chronic stages of the syndrome [40]. Moreover, centrally mediated signs and symptoms become more common in the chronic phases of the condition such as cognitive changes, mood alterations, and motor function disturbances. Thus, we hypothesized that we would observe a shift in the location of mediators driving the CRPS-like changes of our model from the periphery to the spinal cord over time. The fundamental observations of our studies were: (1) cytokines and NGF are elevated in skin at relatively acute time points after tibia fracture and immobilization, but these normalize over time; (2) hindpaw edema, keratinocyte proliferation, and increase in peripheral SP and NK1-receptor expression resolve as the condition becomes more chronic; (3) spinal cord cytokines, NGF, SP, and NK1-receptor levels are increased in the early post-fracture period, and most of these spinal inflammatory changes persist in the chronic post fracture period; (4) spinal cord SP, cytokine, and NGF signaling support nociceptive sensitization at both acute and chronic time points; and (5) mechanical allodynia is the most durable of the CRPS-like changes exhibited by our model over time.

A great deal of attention has been paid to the presence of pain and inflammation-related mediators in the skin of patients with CRPS. Some of the most compelling studies have involved the analysis of fluid from suction blisters made on the skin of CRPS patients. Numerous cytokines including TNFα, IL-6, and others have been discovered at elevated levels [41, 42]. While the relationship of mediator abundance to CRPS duration has not been firmly established, it does appear that cytokine levels may decline over time in some patients [42]. Our study using human hand surgery patients discovered elevated levels of skin cytokines and NGF 3–5 weeks after surgery at the point of cast-removal [43]. Mechanical allodynia, warmth, and edema were all present in these patients suggesting that tissue trauma followed by immobilization may commonly evoke this type of peripheral inflammatory response, though not all patients go on to develop chronic CRPS. Additional studies using a radiolabeled anti-TNF molecule found greatly enhanced TNF activity in the limbs of CRPS patients [44]. On the other hand, results from small human trials using biologic anti-TNF agents are less clear, though encouraging results were noted in some patients [45, 46].

The present study monitored the pathological changes accompanying CRPS-like changes in early (4 weeks) and later (16 weeks) stages of a rat model. By 16 weeks post fracture, vasculature abnormalities, including warmth and edema, recovered as inflammation subsided in skin. Our data indicate that the processes supporting persistent sensitization in the late stage animals are likely spinal. This has implications concerning the tailoring of treatments for early versus late stage disease. Future experiments might exploit the individual patterns of recovery both of humans and laboratory animals to gain insight into the mechanisms contributing to each of the features of CRPS.

Using mouse and rat tibia fracture/cast immobilization models, the effects of peripherally restricted anti-cytokine and anti-NGF have been reported. For example, the administration of the same anti-IL-1ra agent used in these studies (anakinra), the anti-TNF agent etanercept, and an anti-NGF antibody all effectively reduced nociceptive changes [28, 34, 47]. Importantly, all of those studies involved measurements made during the acute phase of the model. Correspondingly, elevated levels of these cytokines and NGF were identified in the skin of the affected limbs. The present group of studies demonstrates that anti-IL-1β and anti-NGF were effective at 4 weeks post fracture, but these agents lost effect at 16 weeks after fracture, despite ongoing nociceptive changes. This corresponded to the resolution of elevated levels of these mediators in the peripheral tissues. On the other hand, our data showed both chronic elevations of IL-1β and NGF in spinal cord tissue, as well as the persistent efficacy of spinally administered IL-1β and NGF inhibitors in reversing nociceptive changes. Thus, the relevant pool of these mediators appears to be located within the CNS in the more chronic phases of the syndrome. We previously demonstrated that keratinocytes are the chief peripheral cell-type producing cytokines and NGF in this model and in CRPS patients [6, 19], though the central sources of these mediators have not been identified. Furthermore, as LY303870, anakinra, and anti-NGF exerted potent anti-nociceptive effects at 4 weeks in the CRPS rat model, an important question is whether these treatments can prevent the development of CRPS when these drugs are administered immediately or very early on after fracture.

Neurogenic inflammation has well-established roles in the peripheral manifestations of CRPS. Substance P (SP) released peripherally from afferent/efferent nerves has been shown to alter capillary permeability in humans, an effect that is exaggerated in CRPS-affected limbs [9] and in animal models of CRPS [16]. These effects are believed to be mediated through increased NK1-receptor expression on endothelial cells in the affected limb [8, 16]. In addition, SP containing fibers penetrate the epidermis. Activation of the NK1 receptors expressed by keratinocytes stimulate these cells to produce cytokines, NGF, and other mediators capable in turn of interacting with receptors on afferent neurons to support nociception [48]. Earlier work demonstrated that administration of the selective NK1 antagonist LY303870 during the early weeks after fracture inhibited nociceptive sensitization, edema, warmth, keratinocyte proliferation, and the up-regulation of cytokines in the skin [17, 18, 25].

Because the fracture/cast rats showed a reduction in hindpaw edema, warmth, keratinocyte proliferation, and skin cytokine levels over time, we hypothesized peripheral SP/NK1 signaling might also diminish over time. Indeed, at 4 weeks post fracture, SP levels in sciatic nerve and NK1-receptor levels in the skin were both elevated. Correspondingly, SP-evoked plasma extravasation was augmented. Both the expression and plasma extravasation responses were similar to those of the control animals by week 16, however. This is additional evidence for a normalization of peripheral neural function and response over time, in spite of persistent nociceptive changes.

In contrast to the expression and functional responses of peripheral tissues, NK1-receptor levels remained elevated in ipsilateral lumbar spinal cord tissue throughout the course of study. Likewise, intrathecal injection of the NK1-receptor antagonist LY303870 reduced nociceptive sensitization at both the 4- and 16-week time points to approximately the same degree (but systemically administered LY303870 appeared to lose analgesic efficacy over time). The NK1 receptor is densely expressed on spinal cord nociceptive projection neurons. Using both pharmacological and neuroablative techniques, it has been shown that spinal NK1-expressing neurons are critical components of nociceptive signaling in many pain models [24, 49, 50]. Though there is some evidence for the local spinal production of SP, spinal NK1 receptors are generally felt to respond to SP released from the central terminals of primary afferent nerves. Thus, central NK1-mediated signaling may be at least partially responsible for the persistent nociceptive sensitization seen in this model. It should be mentioned that NK1-receptor antagonists with good CNS penetrance have not been proven to be effective analgesics in humans, though they do have potent antiemetic properties [51]. We are unaware of a trial of an NK1-receptor antagonist in human CRPS patients, however.

## Conclusions

In these studies, we followed cytokine, neurotrophin, and neuropeptide contributions to the nociceptive and vascular CRPS-like features of a well-characterized fracture/cast model of CRPS. We found evidence of an initially peripherally driven set of processes centralizing to support persistent nociception 16 weeks after the tibia fracture. The gradual loss of the peripheral vascular changes and keratinocyte proliferation, with ongoing nociceptive sensitization, is reminiscent of the transition of warm- to cold-phase CRPS in patients. These observations place emphasis on the targeting of peripheral processes during the acute phases of CRPS, though central processes may be the better targets for chronic CRPS sufferers.

## References

[1] Wasner G, Backonja MM, Baron R (1998). Traumatic neuralgias: complex regional pain syndromes (reflex sympathetic dystrophy and causalgia): clinical characteristics, pathophysiological mechanisms and therapy. Neurol Clin.

[2] Parkitny L, McAuley JH, Di Pietro F, Stanton TR, O'Connell NE, Marinus J, van Hilten JJ, Moseley GL (2013). Inflammation in complex regional pain syndrome: a systematic review and meta-analysis. Neurology.

[3] Linnman C, Becerra L, Borsook D (2013). Inflaming the brain: CRPS a model disease to understand neuroimmune interactions in chronic pain. J Neuroimmune Pharmacol.

[4] Cooper MS, Clark VP (2013). Neuroinflammation, neuroautoimmunity, and the co-morbidities of complex regional pain syndrome. J Neuroimmune Pharmacol.

[5] Li WW, Guo TZ, Shi X, Czirr E, Stan T, Sahbaie P, Wyss-Coray T, Kingery WS, Clark JD (2014). Autoimmunity contributes to nociceptive sensitization in a mouse model of complex regional pain syndrome. Pain.

[6] Birklein F, Drummond PD, Li W, Schlereth T, Albrecht N, Finch PM, Dawson LF, Clark JD, Kingery WS (2014). Activation of cutaneous immune responses in complex regional pain syndrome. J Pain.

[7] Birklein F, O'Neill D, Schlereth T (2015). Complex regional pain syndrome: An optimistic perspective. Neurology.

[8] Leis S, Weber M, Isselmann A, Schmelz M, Birklein F (2003). Substance-P-induced protein extravasation is bilaterally increased in complex regional pain syndrome. Exp Neurol.

[9] Weber M, Birklein F, Neundorfer B, Schmelz M (2001). Facilitated neurogenic inflammation in complex regional pain syndrome. Pain.

[10] Libon DJ, Schwartzman RJ, Eppig J, Wambach D, Brahin E, Peterlin BL, Alexander G, Kalanuria A (2010). Neuropsychological deficits associated with Complex Regional Pain Syndrome. Journal of the International Neuropsychological Society : JINS.

[11] Schilder JC, Schouten AC, Perez RS, Huygen FJ, Dahan A, Noldus LP, van Hilten JJ, Marinus J (2012). Motor control in complex regional pain syndrome: a kinematic analysis. Pain.

[12] Sarangi PP, Ward AJ, Smith EJ, Staddon GE, Atkins RM (1993). Algodystrophy and osteoporosis after tibial fractures. The Journal of bone and joint surgery British volume.

[13] Bickerstaff DR, Kanis JA (1994). Algodystrophy: an under-recognized complication of minor trauma. British journal of rheumatology.

[14] Atkins RM, Duckworth T, Kanis JA (1989). Algodystrophy following Colles' fracture. Journal of hand surgery.

[15] Atkins RM, Kanis JA (1989). The use of dolorimetry in the assessment of post-traumatic algodystrophy of the hand. British journal of rheumatology.

[16] Wei T, Li WW, Guo TZ, Zhao R, Wang L, Clark DJ, Oaklander AL, Schmelz M, Kingery WS (2009). Post-junctional facilitation of Substance P signaling in a tibia fracture rat model of complex regional pain syndrome type I. Pain.

[17] Guo TZ, Offley SC, Boyd EA, Jacobs CR, Kingery WS (2004). Substance P signaling contributes to the vascular and nociceptive abnormalities observed in a tibial fracture rat model of complex regional pain syndrome type I. Pain.

[18] Guo TZ, Wei T, Shi X, Li WW, Hou S, Wang L, Tsujikawa K, Rice KC, Cheng K, Clark DJ (2012). Neuropeptide deficient mice have attenuated nociceptive, vascular, and inflammatory changes in a tibia fracture model of complex regional pain syndrome. Mol Pain..

[19] Li WW, Guo TZ, Li XQ, Kingery WS, Clark JD (2010). Fracture induces keratinocyte activation, proliferation, and expression of pro-nociceptive inflammatory mediators. Pain.

[20] Wesseldijk F, Huygen FJ, Heijmans-Antonissen C, Niehof SP, Zijlstra FJ (2008). Six years follow-up of the levels of TNF-alpha and IL-6 in patients with complex regional pain syndrome type 1. Mediators Inflamm..

[21] Zimmermann M (1983). Ethical guidelines for investigations of experimental pain in conscious animals. Pain.

[22] Gitter BD, Bruns RF, Howbert JJ, Waters DC, Threlkeld PG, Cox LM, Nixon JA, Lobb KL, Mason NR, Stengel PW (1995). Pharmacological characterization of LY303870: a novel, potent and selective nonpeptide substance P (neurokinin-1) receptor antagonist. J Pharmacol Exp Ther.

[23] Hipskind PA, Howbert JJ, Bruns RF, Cho SS, Crowell TA, Foreman MM, Gehlert DR, Iyengar S, Johnson KW, Krushinski JH (1996). 3-Aryl-1,2-diacetamidopropane derivatives as novel and potent NK-1 receptor antagonists. J Med Chem.

[24] Iyengar S, Hipskind PA, Gehlert DR, Schober D, Lobb KL, Nixon JA, Helton DR, Kallman MJ, Boucher S, Couture R (1997). LY303870, a centrally active neurokinin-1 antagonist with a long duration of action. J Pharmacol Exp Ther.

[25] Wei T, Guo TZ, Li WW, Hou S, Kingery WS, Clark JD (2012). Keratinocyte expression of inflammatory mediators plays a crucial role in substance P-induced acute and chronic pain. J Neuroinflammation..

[26] Opp MR, Krueger JM (1991). Interleukin 1-receptor antagonist blocks interleukin 1-induced sleep and fever. Am J Physiol.

[27] Kimble RB, Vannice JL, Bloedow DC, Thompson RC, Hopfer W, Kung VT, Brownfield C, Pacifici R (1994). Interleukin-1 receptor antagonist decreases bone loss and bone resorption in ovariectomized rats. J Clin Invest.

[28] Li WW, Sabsovich I, Guo TZ, Zhao R, Kingery WS, Clark JD (2009). The role of enhanced cutaneous IL-1beta signaling in a rat tibia fracture model of complex regional pain syndrome. Pain.

[29] Wolf G, Livshits D, Beilin B, Yirmiya R, Shavit Y (2008). Interleukin-1 signaling is required for induction and maintenance of postoperative incisional pain: genetic and pharmacological studies in mice. Brain Behav Immun.

[30] Hongo JS, Laramee GR, Urfer R, Shelton DL, Restivo T, Sadick M, Galloway A, Chu H, Winslow JW (2000). Antibody binding regions on human nerve growth factor identified by homolog- and alanine-scanning mutagenesis. Hybridoma.

[31] Halvorson KG, Kubota K, Sevcik MA, Lindsay TH, Sotillo JE, Ghilardi JR, Rosol TJ, Boustany L, Shelton DL, Mantyh PW (2005). A blocking antibody to nerve growth factor attenuates skeletal pain induced by prostate tumor cells growing in bone. Cancer Res.

[32] Sevcik MA, Ghilardi JR, Peters CM, Lindsay TH, Halvorson KG, Jonas BM, Kubota K, Kuskowski MA, Boustany L, Shelton DL (2005). Anti-NGF therapy profoundly reduces bone cancer pain and the accompanying increase in markers of peripheral and central sensitization. Pain.

[33] Shelton DL, Zeller J, Ho WH, Pons J, Rosenthal A (2005). Nerve growth factor mediates hyperalgesia and cachexia in auto-immune arthritis. Pain.

[34] Sabsovich I, Wei T, Guo TZ, Zhao R, Shi X, Li X, Yeomans DC, Klyukinov M, Kingery WS, Clark JD (2008). Effect of anti-NGF antibodies in a rat tibia fracture model of complex regional pain syndrome type I. Pain.

[35] Guo TZ, Wei T, Kingery WS (2006). Glucocorticoid inhibition of vascular abnormalities in a tibia fracture rat model of complex regional pain syndrome type I. Pain.

[36] Kingery WS, Davies MF, Clark JD (2003). A substance P receptor (NK1) antagonist can reverse vascular and nociceptive abnormalities in a rat model of complex regional pain syndrome type II. Pain.

[37] Harden RN, Bruehl S, Stanton-Hicks M, Wilson PR (2007). Proposed new diagnostic criteria for complex regional pain syndrome. Pain Med.

[38] Birklein F, Schmelz M (2008). Neuropeptides, neurogenic inflammation and complex regional pain syndrome (CRPS). Neurosci Lett.

[39] Bruehl S (2010). An update on the pathophysiology of complex regional pain syndrome. Anesthesiology.

[40] Gallagher JJ, Tajerian M, Guo T, Shi X, Li W, Zheng M, Peltz G, Kingery WS, Clark JD (2013). Acute and chronic phases of complex regional pain syndrome in mice are accompanied by distinct transcriptional changes in the spinal cord. Mol Pain..

[41] Munnikes RJ, Muis C, Boersma M, Heijmans-Antonissen C, Zijlstra FJ, Huygen FJ (2005). Intermediate stage complex regional pain syndrome type 1 is unrelated to proinflammatory cytokines. Mediators Inflamm.

[42] Lenz M, Uceyler N, Frettloh J, Hoffken O, Krumova EK, Lissek S, Reinersmann A, Sommer C, Stude P, Waaga-Gasser AM (2013). Local cytokine changes in complex regional pain syndrome type I (CRPS I) resolve after 6 months. Pain.

[43] Pepper A, Li W, Kingery WS, Angst MS, Curtin CM, Clark JD (2013). Changes resembling complex regional pain syndrome following surgery and immobilization. J Pain.

[44] Bernateck M, Karst M, Gratz KF, Meyer GJ, Fischer MJ, Knapp WH, Koppert W, Brunkhorst T (2010). The first scintigraphic detection of tumor necrosis factor-alpha in patients with complex regional pain syndrome type 1. Anesth Analg.

[45] Huygen FJ, Niehof S, Zijlstra FJ, van Hagen PM, van Daele PL (2004). Successful treatment of CRPS 1 with anti-TNF. J Pain Symptom Manage.

[46] Dirckx M, Groeneweg G, Wesseldijk F, Stronks DL, Huygen FJ (2013). Report of a preliminary discontinued double-blind, randomized, placebo-controlled trial of the anti-TNF-alpha chimeric monoclonal antibody infliximab in complex regional pain syndrome. Pain Pract.

[47] Sabsovich I, Guo TZ, Wei T, Zhao R, Li X, Clark DJ, Geis C, Sommer C, Kingery WS (2008). TNF signaling contributes to the development of nociceptive sensitization in a tibia fracture model of complex regional pain syndrome type I. Pain.

[48] Shi X, Wang L, Clark JD, Kingery WS (2013). Keratinocytes express cytokines and nerve growth factor in response to neuropeptide activation of the ERK1/2 and JNK MAPK transcription pathways. Regul Pept..

[49] Castro AR, Pinto M, Lima D, Tavares I (2006). Secondary hyperalgesia in the monoarthritic rat is mediated by GABAB and NK1 receptors of spinal dorsal horn neurons: a behavior and c-fos study. Neuroscience.

[50] Campbell EA, Gentry CT, Patel S, Panesar MS, Walpole CS, Urban L (1998). Selective neurokinin-1 receptor antagonists are anti-hyperalgesic in a model of neuropathic pain in the guinea-pig. Neuroscience.

[51] Patel L, Lindley C (2003). Aprepitant--a novel NK1-receptor antagonist. Expert Opin Pharmacother.

